# Issues in Delivering Morbidity Management for Lymphatic Filariasis Elimination: A Study in Pondicherry, South India

**DOI:** 10.1100/2012/372618

**Published:** 2012-04-30

**Authors:** A. Krishna Kumari, Yuvaraj J, L. K Das

**Affiliations:** ^1^Vector Control Research Centre, Indian Council of Medical Research, Medical Complex, Indira Nagar, Pondicherry 605 006, India; ^2^National Institute of Epidemiology, Indian Council of Medical Research, Tamil Nadu Housing Board, Ayapakkam, Chennai 600077, India

## Abstract

Lymphatic filariasis is a vector borne parasitic disease causing long term disability. The Global Programme to Eliminate Lymphatic Filariasis aims to achieve its objective through two strategies; Mass Drug Administration (MDA) to interrupt transmission and Morbidity Management (MM) to manage disability for those already affected. MDA is going on in full swing in endemic areas; but MM is lagging behind. An exploratory study was conducted in Pondicherry through focus group discussions to find out whether there are delivery issues if any, in the MM programme and get suggestions from end users. The study results show that MM has not received the same attention as MDA and there are shortcomings in the delivery mechanism of the programme. The importance of these findings are discussed and suggestions given for improving the programme.

## 1. Introduction

Lymphatic filariasis (LF) a vector-borne, chronically disabling parasitic infection causing elephantiasis, lymphoedema, and hydrocele, is a major public health problem [1, 2] as well as a serious socioeconomic problem due to its morbid condition, social stigma, and considerable economic loss in many developing countries [3, 4], and this disease has been ranked as the second leading cause of long-term chronic disability worldwide [5]. The disability caused renders those afflicted unproductive and unable to contribute to national and their individual economic progress [6]. In the year 1997, following advances in diagnosis and treatment, the World Health Organization (WHO) identified lymphatic filariasis as potentially eradicable or eradicable, leading to the launching of the Global Programme to Eliminate Lymphatic Filariasis (GPELF) in the year 2000 [6, 7]. According to the Global Alliance to Eliminate Lymphatic Filariasis (GAELF), a public-private partnership was created in 2000 to assist in advocacy, resource mobilisation, and programme implementation. As there are many different presentations of clinical disease related to LF, there is no single drug or treatment that is effective for all cases, and therefore three issues should be considered for all patients (1) antiparasitic drug therapy, (2) supportive clinical care, and (3) patient education and counseling [8]. Thus the LF elimination programme aims to achieve its objective using a twofold strategy of interrupting transmission with repeated annual rounds of Mass Drug Administration (MDA) and Morbidity Management (MM) for those already infected, to prevent/manage disabilities [9]. 

As a signatory to 50th World Health Assembly resolution on global elimination of lymphatic filariasis, India launched a revised filariasis control program in 1997 [10]. Accordingly, the National Health Policy 2002 set its aim to eliminate lymphatic filariasis by 2015 and entrusted the task to the National Vector Borne Disease Control Programme (NVBDCP), the central nodal agency for the prevention and control of vector borne diseases in the country. The NVBDCP aims to achieve this objective using the strategy of MDA of anti filarial drugs (diethylcarbamazine (DEC)+Albendazole), for 5 years or more to the population excluding children below two years, pregnant women, and seriously ill persons in affected areas to interrupt transmission of disease and self management of lymphoedema with limb hygiene [11] and hydrocelectomy operations in identified Community Health Centres (CHCs) and hospitals [12]. Of the twin components of GPELF, MDA is in operation through national programs in 51 of the world's 81 LF endemic countries, but MM has been initiated only in 27 out of the 81 endemic countries [13]. About 40 million people who already have the disease are yet to be covered by the programme [14]. Since providing care for persons who suffer from the major forms of filariasis-related morbidity is a major goal of GPELF [15], and because recent studies offer hope to chronic LF patients by proving that, by following the simple lymphoedema management measures, morbidity due to LF can be reduced within a period as short as 4.5 months [16], it is important to address the problems affecting this programme.

This study is an attempt to explore whether there are delivery issues if any that are dragging the programme behind and get suggestions from end users to get maximum benefit from the programme. The study specifically examined three aspects: awareness of the MM programme, practice of MM, and benefits gained from the MM programme. It is intended that this research will provide information necessary to address the loopholes and provide suggestions to make the programme responsive to the need of LF patients.

## 2. Methods

### 2.1. Study Settings

The study was conducted in the months of April-May 2009, at two Anganwadis (Anganwadis are government-sponsored child-care centres serving as day care and preschools for children of ages 0–6). in Kombakkam (rural area) and Mudaliarpet (urban area) coming within the limits of Murugampakkam Primary Health Centre; population 25768 and Mudaliarpet Primary Health Centre; population 53381 [17], respectively, in Pondicherry region in the Union Territory of Puducherry, South India. Pondicherry has an area of 290 km^2^ and a population of 7,35,332 [18]. The mean maximum temperature is 38.2°C, and mean minimum temperature is 24°C [19]. The study area is known to be endemic for filariasis [20]. Men and women diagnosed with chronic lymphoedema due to lymphatic filariasis, living in the two study areas, and belonging to the age group of 15–60, were purposively selected from the list of filariasis patients identified through line listing by the State Filaria Control Unit (SFCU) of Pondicherry for morbidity management, as part of the national programme for elimination of filariasis for the study. Purposive sampling helped to ensure diversity in terms of age, marital status, education, rural/urban difference, and socioeconomic background of participants. Majority of the study participants in the rural area were agricultural labourers, and others included flower vendors, house wives, and people who did not perform any occupation. Among the participants from the urban area, majority were casual labourers; some were daily wage workers in private companies, some were retired persons, some were house wives, and one was a priest. Majority of the participants from the rural area did not have formal education where as majority from the urban area had formal education ranging from primary level to high school level. 

### 2.2. Study Designs and Data Collection

This study was exploratory in nature, and data was collected through focus group discussion method. Eight focus group discussions (four for men and four for women in two PHC areas) were conducted with 6–10 persons in each group. The discussions lasted from 60 to 80 minutes and were held around 6 O'clock in the evening in the premises of two Anganwadis in the locality, a convenient place for local people to assemble in the evening after a day's work. All the discussions were conducted following the standard procedures for conducting focus group discussions consisting of a team with a moderator to guide the discussion, a rapporteur to record the discussion, and two assistants for general arrangements. A sociologist with research and field experience moderated the discussions.

The study had the approval of the Vector Control Research Centre's (VCRC) Institutional Human Ethics Committee, and informed written consent was obtained from each participant who took part in the focus group discussions. The participants were informed that the discussion may last approximately one hour and that, the team will be taking notes and tape recording the discussion, so that whatever the participants said would not be missed. Participation was voluntary and confidentiality of the opinions raised by the participants was assured. The discussions were audiotaped as well as written down by the rapporteur. The discussion focused on aspects related to present treatment source, reasons for opting that particular source of treatment, awareness of the MM Programme, their practice of leg hygiene, improvement due to leg hygiene, opinion about the programme, suggestion if any for improvement of the programme, and change in perception about leg hygiene after attending the focus group discussion.

### 2.3. Data Analysis

The conversations during the focus group discussions were translated and transcribed into English and analysed for the trends and patterns that emerged from the discussions. The awareness of MM, practice of MM, and benefit gained from MM were identified and given codes. The codes were then categorised, and the emerging themes were found out.

## 3. Results

The following themes emerged from the focus group discussions

### 3.1. Specific Themes Related to the Morbidity Management Programme

- LF patients were not aware of morbidity management programme.
- Clinicians and health workers did not seem to be aware of the new strategies of morbidity management.
- Morbidity management programme lacked adequate publicity in endemic areas.
- Leg hygiene demonstration camps were conducted without publicity and went unnoticed by patients.
- Training on leg hygiene was given only to a small proportion of the identified cases.
- The camps demonstrated leg hygiene but failed to effectively communicate its benefits.
- Only those who knew about the benefits of leg hygiene through some source practiced it; others considered washing the leg during bath was sufficient.
- Those who practiced leg hygiene-reported signs of improvement.

### 3.2. Other Themes Related to Filariasis Morbidity

- Restricted mobility due to oedema and pain affects treatment seeking.
- Due to problems in mobility, patients hesitate to avail treatment from distant and crowded places
- Patients in the urban area considered the SFCU clinic at Saram a very convenient treatment source for them. They could take regular treatment because of the SFCU clinic.
- LF patients in rural areas found it difficult to access the SFCU clinic Therefore, they took treatment only during ADL attacks.
- Chronic lymphoedema patients, who did not perceive any improvement in their condition even after prolonged treatment, gradually stopped treatment.
- Perception of the participants regarding the importance of leg hygiene changed after the focus group discussion.

### 3.3. Patient's Suggestion Regarding Morbidity Management Programme

The patients were asked to give their suggestions for improving the MM programme. The following were their suggestions.

- MM programme should be given wide publicity and advertisement.
- Leg hygiene and exercises should be demonstrated with prior notice at more accessible places like PHCs or Anganwadis, at a convenient time for casual labourers and agricultural workers who leave their homes early in the morning and return late in the evening.
- Health talk should be given by an expert before demonstrating leg hygiene, emphasizing its importance in controlling ADL attacks and slowing down disease progression.
- Not only chronic cases, patients with lower grades of lymph oedema also should be covered under the programme and made aware of the importance of leg hygiene in controlling ADL attacks and slowing down disease progression.
- A relative of the patient may also be permitted to attend the leg hygiene demonstration camp so that the family members can motivate or help the patient in maintaining leg hygiene.

## 4. Results in Detail

The themes that emerged from the study are described below.

### 4.1. Patients Were Not Aware of the Morbidity Management Programme

Participants in the urban area as well as the rural area were not aware of the MM programme nor the camps meant for demonstrating leg hygiene and exercises to the patients. Clinicians and health workers also did not seem to be aware of the new strategies of morbidity management for LF. This was evident from the fact that clinicians and health workers gave only a routine advice to keep the legs clean, as reported by the patients.

### 4.2. Morbidity Management Camps Failed to Convey the Message That Leg Hygiene Could Prevent ADL Attacks and Control Disease Progression

Among the participants from the urban and rural areas, only one person from the urban area had attended a morbidity management demonstration camp organised by the PHC in the locality, to promote leg hygiene. Though he attended the demonstration camp, he got the impression that it was just another routine advice for maintaining cleanliness and did not consider it very important to practice it. The camp could not convey the idea that leg hygiene can prevent ADL attacks and control disease progression. Therefore, demonstration classes should be planned and organised in consultation with experts, well conversant in communicating with the community. 

### 4.3. Morbidity Management Programme Lacked Adequate Publicity

It was understood that publicity about MM was limited to house visits by health workers. There were no posters, banners, or announcements with public addressing systems. The filariasis patients were mostly from poor economic background, performing agricultural activities, or engaged as casual labourers. These labourers leave their homes early in the morning and return late in the evening. Their houses would be locked during day time when the health workers visit their houses. Information passed through house visits was not sufficient to reach all the LF patients.

### 4.4. LF Patients Considered that Cleaning the Legs during Daily Bath Was Sufficient

While discussing about their current practice of leg hygiene, majority of the participants said that they washed their legs when they took bath and considered that, was enough. The participants regularly attending the SFCU clinic said that, the chronic patients with higher grades of lymphoedema were advised to keep their legs clean. But they were not told anything about the importance of leg hygiene in preventing ADL attacks or slowing down the disease progression.

### 4.5. Some Patients Knew the Benefit of Leg Hygiene from Other Sources

Though majority of the participants from the urban as well as rural areas were not aware of MM programme, there were some respondents from the urban area who knew about leg hygiene and simple exercises for LF. It was understood that they were participants of a research project of VCRC to assess the impact of leg hygiene on lymphoedema volume, skin thickness, and physical disability in patients with filarial lymphoedema. These patients were trained in leg hygiene according to standard guidelines.

### 4.6. Those Who Practiced Limb Hygiene Reported Improvement

Though very visible changes could not be observed in the size of the oedema within a short period, the few who were practicing leg hygiene for the past one year reported changes which seem to be encouraging and could be clear signs of improvement in the long run. Frequency of ADL had reduced, the skin had become soft, flexibility of limbs had improved, and domestic activities had become easier for them.

### 4.7. Restricted Mobility due to the Disease Affects Treatment

Higher grades of lymphoedema and hydrocele considerably restrict the mobility of lymphatic filariasis patients. They have difficulty in walking long distances, climbing staircases, riding bicycles, or travelling in the crowded public transport systems to reach treatment centres, wait there in long queues, and avail timely treatment. For some patients, the first occurrence of lymphoedema itself was associated with a long-distance travel. Policy makers and programme managers may consider the restricted mobility factor of LF patients seriously in any future course of action for LF patients.

### 4.8. Filariasis Clinics Are the Preferred Treatment Source but Are Not Accessible to All LF Patients

Government hospitals are always crowded and necessitate the patients to stand for long hours in queues to see the doctor and get medicines. For an LF patient, continuous standing triggers off pain and increase in oedema. Private hospitals are too expensive, and majority of the LF patients who are from poor economic background cannot afford to seek treatment there. Because of these reasons, LF patients prefer to seek treatment from filariasis clinics. But in Pondicherry there are only two filariasis clinics, one is the filariasis clinic run by the SFCU, Pondicherry, and the other is the biweekly filariasis clinic run by the VCRC, Pondicherry. The SFCU clinic is situated at Saram in the city, and VCRC clinic is situated at Gorimedu, a place 5 km away from the city near the Chennai-Dindivanam road. LF patients in the city make use of these facilities, but patients in rural areas find it difficult to reach them. Provision of more filariasis clinics in rural areas or providing filariasis clinics attached to the PHCs may help the LF patients to access timely treatment.

### 4.9. Patients in Urban Area Took Regular Treatment While Patients in Rural Areas Took Treatment Only during ADL Attack

In order to prevent ADL attacks and disease progression, LF patients are advised to be on regular treatment. The SFCU clinic provides medicines for one month and advise the patient to follow up the treatment every month. People in the urban area were taking regular treatment for filariasis. They took treatment either from the SFCU clinic or the biweekly filariasis clinic run by the VCRC or from both. Those patients who could not visit either of these clinics resorted to self-treatment with old prescriptions.

Patients in the rural area took treatment only during ADL attacks. Whenever there was an ADL attack, they took treatment from the nearby private clinics. The SFCU clinic and VCRC clinic were less accessible to these patients since both were located in the city and functioned on specific days in a week.

### 4.10. Patients, Who Did Not Perceive Any Improvement in Their Condition, Stopped Treatment

Patients who did not perceive any improvement in their condition in one system of medicine switched over to other systems of medicine. When they did not find any improvement in other systems also, they finally gave up treatment altogether.

### 4.11. Change in Perception of Leg Hygiene

At the end of each discussion the group members were asked whether their perception of the need for leg hygiene had changed after participating in the focus group discussion. All members said that the discussions were very useful. Many respondents said that, after attending the focus group discussion only, they came to know how important was leg hygiene and simple foot exercises for an LF patient.

## 5. Discussion

Research during the past two decades has thrown more light into the morbidity aspects of lymphatic filariasis and paved the way for the inclusion of MM as one of the components of the GPELF (TDR, 2005).

However, the study results show that, of the two components of GPELF, the morbidity management component is lagging behind MDA, and there are shortcomings in the delivery mechanism of the programme. It was found that patients were not aware of the programme. From the accounts of the patients, it is understood that, other than a routine, general advice to keep their legs clean, the clinicians or the health workers did not inform the patients about the new strategies of leg hygiene. It should be assumed that the clinicians and health workers are also not aware of the same.

A study on clinician's practices related to management of filarial adenolymphangitis and lymphoedema in Orissa has also reported that none of the clinicians interviewed during their study instantly revealed that they give advice on foot hygiene as part of lymphoedema management [21]. Another study on lymphoedema care in Orissa has reported that the health workers in the PHCs have not received any training on lymphedema care, except a half-day training session on drug distribution before the yearly mass drug administration of the GPELF. Since there were no facilities in the PHCs to take care of lymphedema patients, the staff were instructed to refer them to any hospital [22]. A study in Srilanka has also reported that neither limb-care methods nor exercise had been recommended by any clinician, to any of the patients they interviewed for their study and that patients who do not find any improvement in their condition gradually stopped taking treatment for filariasis [23]. A study in Madhya Pradesh, to review the progress of Mass Drug Administration of single dose of di-ethyl-carbamazine (DEC), has reported that the level of awareness of the morbidity management in the community was low, and very few subjects with LF, who were interviewed, could answer the proper method of care. They observed that training on morbidity management was given to only a small proportion of the identified cases [24].

Though some shortcomings have come to light in the delivery mechanism of the morbidity management programme, they can be rectified with proper planning and cooperation. For instance, since the PHCs have been assigned the charge of morbidity management, it is logical to enquire whether the PHCs have the necessary facilities, trained man power, and additional time to spare apart from its routine works like immunization, prevention of malnutrition, care during pregnancy, child birth, postnatal care, treatment of common illnesses, and so forth. If the PHCs do not have the above, the feasibility of opening filariasis clinics attached to the PHCs in rural areas by the SFCU may be considered as one of the option. The medical personnel attending the LF patients in the PHCs and the SFCU may be given opportunity to learn about new and developing areas in the field of lymphatic filariasis through Continued Medical Education (CME). Health workers in the PHCs and SFCU clinics need to be made aware of the importance of leg hygiene in preventing ADL attacks and there by slowing down the progression of lymphoedema. They need to be trained to demonstrate leg hygiene and exercises to the patients. Lack of publicity and advertisement is an important lacuna of the MM programme. Experts in the field of communication and preparation of Information Education & Communication (IEC) tools should be consulted and involved while preparing suitable IEC materials for LF patients who are generally from poor back grounds and are less educated.

 The fact that, even after prolonged treatment, many LF patients do not perceive any improvement in their condition, lead us to the assumption that intake of medicines alone is not sufficient to improve the condition of the patient, but leg hygiene and simple exercises are also essential for improving the quality of life of the patients. This underlines the need to consider morbidity management as an important component of the Global Programme to eliminate lymphatic filariasis.

## Abbreviations

LF: Lymphatic filariasis.
WHO: World Health Organization
GPELF: Global Programme to Eliminate Lymphatic Filariasis
GAELF: Global Alliance to Eliminate Lymphatic Filariasis
MDA: Mass Drug Administration
MM: Morbidity management
NVBDCP: National Vector Borne Disease Control Programme
DEC: Diethylcarbamazine citrate
CHCs: Community Health Centres
PHC: Primary Health Centre
SFCU: State Filaria Control Unit
VCRC: Vector Control Research Centre
ADL: Adenolymphangitis
CME: Continued Medical Education
IEC: Information Education & Communication
TDR: Tropical Disease Research (WHO).

## References

[1] Pani SP, Srividya A (1995). Clinical manifestations of bancroftian filariasis with special reference to lymphoedema grading. *Indian Journal of Medical Research*.

[2] Ottesen EA, Duke BO, Karam M, Behbehani K (1997). Strategies and tools for the control/elimination of lymphatic filariasis. *Bulletin of the World Health Organization*.

[3] Mukhopadhyay AK (2010). Lymphatic filariasis in Andhra Pradesh Paper Mill Colony, Rajahmundry, India after nine rounds of MDA programme. *Journal of Vector Borne Diseases*.

[4] Ramaiah KD, Guyatt H, Ramu K, Vanamail P, Pani SP, Das PK (1999). Treatment costs and loss of work time to individuals with chronic lymphatic filariasis in rural communities in South India. *Tropical Medicine and International Health*.

[5] World Health Organization (1995). *The World Health Report*.

[6] Mathieu E, Amann J, Eigege A, Richards F, Sodahlon Y (2008). Collecting baseline information for national morbidity alleviation programs: different methods to estimate lymphatic filariasis morbidity prevalence. *American Journal of Tropical Medicine and Hygiene*.

[7] WHO Neglected tropical diseases. http://www.who.int/neglected_diseases/preventive_chemotherapy/5gaelf_speech_full/en/index.html.

[8] GAELF A future free of LF. http://www.filariasis.org/all_about_lf/management.html.

[9] Das PK, Ramaiah KD (2002). Entomological monitoring of annual mass drug administrations for the control or elimination of lymphatic filariasis. *Annals of Tropical Medicine and Parasitology*.

[10] Agrawal (2006). Lymphatic filariasis in India: problems, challenges and new initiatives. *Medical Journal Armed Forces India*.

[11] Vaqas B, Ryan TJ (2003). Lymphoedema: pathophysiology and management in resource-poor settings—relevance for lymphatic filariasis control programmes. *Filaria Journal*.

[12] NVBDCP Strategy for elimination of lymphatic filariasis. http://nvbdcp.gov.in/fil8.html.

[13] http://www.who.int/wer/2009/wer8442/en/index.html.

[14] Perera M, Whitehead M, Molyneux D, Weerasooriya M, Gunatilleke G (2007). Neglected patients with a neglected disease? A qualitative study of lymphatic filariasis. *PLoS Neglected Tropical Diseases*.

[15] Addiss DG (2005). Global elimination of lymphatic filariasis: origins, progress and challenges. *Indian Journal of Urology*.

[16] Jullien P, Somé JD, Brantus P, Bougma RW, Bamba I, Kyelem D (2011). Efficacy of home-based lymphoedema management in reducing acute attacks in subjects with lymphatic filariasis in Burkina Faso. *Acta Tropica*.

[17] Puducherry Government Health facilities at a glance. http://health.puducherry.gov.in/.

[18] Puducherry Government Plan Formulation Document. http://pandr.puducherry.gov.in/Plan%20Formulation/Plan%20Document/DAP%202011-12/INDEX.htm.

[19] Puducherry Government Department of Tourism. Welcome to Puducherry. http://tourism.pondicherry.gov.in/pdf/pre-Inv.ppt.

[20] Das PK, Monoharan A, Subramanian S (1992). Bancroftian filariasis in Pondicherry, South India-epidemiological impact of recovery of the vector population. *Epidemiology and Infection*.

[21] Kerketta AS, Babu BV, Swain BK (2007). Clinicians practices related to management of filarial adenolymphangitis and lymphoedema in Orissa, India. *Acta Tropica*.

[22] Rath K, Swain BK, Mishra S, Patasahani T, Kerketta AS, Babu BV (2005). Peripheral health workers’ knowledge and practices related to filarial lymphedema care: a study in an endemic district of Orissa, India. *American Journal of Tropical Medicine and Hygiene*.

[23] Yahathugoda TC, Wickramasinghe D, Weerasooriya MV, Samarawickrema WA (2005). Lymphoedema and its management in cases of lymphatic filariasis: the current situation in three suburbs of Matara, Sri Lanka, before the introduction of a morbidity-control programme. *Annals of Tropical Medicine and Parasitology*.

[24] Lahariya C, Mishra A (2008). Strengthening of mass drug administration implementation is required to eliminate lymphatic filariasis from India: an evaluation study. *Journal of Vector Borne Diseases*.

